# Implicaţii psihosociale ale scăderii în greutate

**Published:** 2014

**Authors:** Ladea Maria, Popa Teodora Alexandra, Bran Mihai

**Affiliations:** * Spitalul Clinic de Psihiatrie “Prof. Dr. Al. Obregia”, București, România, Spitalul Clinic de Urgență “Sf. Ioan”, București, România

**Rezumat**

Multe dintre rezultatele aşteptate în urma scăderii în greutate sunt de natură psihosocială, precum ameliorarea dispoziţiei, a calităţii vieţii şi a imaginii de sine. Schimbările pozitive în starea psihosocială secundare pierderii în greutate, se aplică atât la persoanele obeze din populaţia generală, cât şi la persoanele care sunt doar ușor supraponderale (<20% peste greutatea lor recomandată). Studiile arată că scăderi semnificative în greutate determină ameliorări importante ale calităţii vieţii.

Unii indivizi experimentează emoţii negative ca urmare a recâştigării în greutate, acestea fiind un obstacol în evoluţia favorabilă.

În ciuda prevalenţei crescute a recâştigării în greutate, există puţine date despre efectele sale psihologice. Scăderea în greutate este asociată cu îmbunătăţirea imaginii de sine, însă lipsa unei relaţii direct proportionale între cele două şi afectarea parţială dată de o minimă recâştigare în greutate sugerează că scăderea în greutate nu ar trebui să fie principala metodă de îmbunătăţire a imaginii de sine.

Efectul pozitiv al terapiei comportamentale a fost confirmat, subiecţii trataţi prin dietă asociat cu terapie comportamentală beneficiind de o ameliorare semnificativă a dispoziţiei faţă de cei care au apelat doar la dietă.

Deşi milioane de oameni apelează la diete, nu se cunoaşte foarte bine dacă obţin rezultatele psihosociale dorite. Studiile arată consecinţele pozitive ale obţinerii unei greutăţi normale, însă nu există multe date despre efectele negative ale eşecului de a slăbi sau de a menţine greutatea.

**Cuvinte-cheie:** psihosocial, scădere în greutate, calitatea vieţii

